# Involvement of a phospholipase C in the hemolytic activity of a clinical strain of *Pseudomonas fluorescens*

**DOI:** 10.1186/1471-2180-8-189

**Published:** 2008-10-30

**Authors:** Gaelle Rossignol, Annabelle Merieau, Josette Guerillon, Wilfried Veron, Olivier Lesouhaitier, Marc GJ Feuilloley, Nicole Orange

**Affiliations:** 1 Laboratory of Cold Microbiology, UPRES EA 4312, University of Rouen, 55 rue Saint Germain, 27000 Evreux, France

## Abstract

**Background:**

*Pseudomonas fluorescens *is a ubiquitous Gram-negative bacterium frequently encountered in hospitals as a contaminant of injectable material and surfaces. This psychrotrophic bacterium, commonly described as unable to grow at temperatures above 32°C, is now considered non pathogenic. We studied a recently identified clinical strain of *P. fluorescens *biovar I, MFN1032, which is considered to cause human lung infection and can grow at 37°C in laboratory conditions.

**Results:**

We found that MFN1032 secreted extracellular factors with a lytic potential at least as high as that of MF37, a psychrotrophic strain of *P. fluorescens *or the mesophilic opportunistic pathogen, *Pseudomonas aeruginosa *PAO1. We demonstrated the direct, and indirect – through increases in biosurfactant release – involvement of a phospholipase C in the hemolytic activity of this bacterium. Sequence analysis assigned this phospholipase C to a new group of phospholipases C different from those produced by *P. aeruginosa*. We show that changes in PlcC production have pleiotropic effects and that *plcC *overexpression and *plcC *extinction increase MFN1032 toxicity and colonization, respectively.

**Conclusion:**

This study provides the first demonstration that a PLC is involved in the secreted hemolytic activity of a clinical strain of *Pseudomonas fluorescens*. Moreover, this phospholipase C seems to belong to a complex biological network associated with the biosurfactant production.

## Background

*Pseudomonas fluorescens *is a ubiquitous Gram-negative bacterium frequently encountered in the environment. Naturally resistant to a wide range of antibiotics and disinfectants, this bacterium is commonly found in hospitals as a contaminant of cleaning solutions and even injectable material [1]. This bacterium is characterized by its very large genome, containing a large number of regulatory genes conferring a very high potential to adapt to environmental variation [2]. *P. fluorescens *is generally considered psychrotrophic species, but the presence of this bacterium in hospitals has long been demonstrated [3]. The broad clinical spectrum of this bacterium, which may colonize the airways [4], urinary tract [5] and blood [6] of immunocompromised patients and previously healthy patients, demonstrates that human body temperature is not necessarily a barrier to the development of this microorganism. These findings strongly suggest that different strains have evolved to deal with this specific environment [7]. Previous investigations of the infectious potential of *P. fluorescens *have demonstrated that this bacterium can bind specifically to the cytoplasmic membrane of neurons and glial cells [8]. Attachment to the cytoplasmic membrane of the host cell is associated with the induction of apoptosis and necrosis [9]. LPS has clearly been implicated in cytotoxicity, but other factors released along with LPS during cytoadhesion also seem to be essential for the virulence of this bacterium [10]. The virulence factors of *Pseudomonas aeruginosa *and *Burkholderia *sp. have been studied in detail, but little is known about those of *P. fluorescens *[11,12]. *P. fluorescens *bacteria synthesize and release various extracellular enzymes, including a protease [13] and a lipase [14], which may be involved in virulence. They also produce phospholipase C (PLC) [15-19] – an enzyme produced by many bacterial pathogens and associated with various levels of virulence [20]. The pathophysiological role of secreted PLCs depends on bacterial species, extending from that of a major virulence factor to a minor metabolic factor involved in bacterial survival or dissemination only, without cytotoxic effects [21,22]. However, the effects of PLCs may be more subtle. PLC interferes with eukaryotic cellular signaling cascades and seems to be able to modulate the host immune response [21]. Several members of the *Pseudomonas *family produce PLCs [21], and the PLCs synthesized by *P. aeruginosa *have been studied in detail [23]. The virulence potential of the PLCs of this bacterium has often been associated with hemolytic activity [24]. Conversely, our knowledge about hemolysin production by *P. fluorescens *remains very limited. The PLCs identified in this species to date have essentially been studied biochemically, without considering their infectious potential.

We studied a recently identified strain of *P. fluorescens *(MFN1032) [7] with mesophilic behavior and hemolytic activity, comparing the cytotoxicity of the factors secreted by this clinical strain with those secreted by the environmental *P. fluorescens *strain MF37. We also included the opportunistic pathogen *P. aeruginosa *in the study, as a reference. We identified a phospholipase C (PlcC) from MFN1032 and compared its amino-acid sequence with those of the PLCs produced by other *P. fluorescens *species and other Gram-negative and Gram-positive bacteria. We then evaluated the involvement of PlcC in the hemolytic activity of MFN1032, by constructing *plcC *mutants.

## Results

### Characterization of the MFN1032 strain

**Figure 1:**
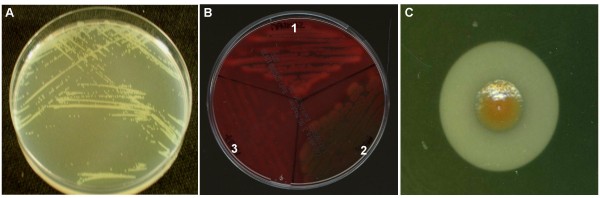
**Growth and extracellular activities of MFN1032**. **A- **Growth of MFN1032 on LB agar plates after 24 hours at 37°C. **B- **Secreted hemolytic activity of MFN1032 on sheep red blood agar plate after 48 hours of incubation at 28°C (1), and comparison with PAO1 (2) and MF37 (3). **C**- MFN1032 lecithinase activity on egg-yolk agar plate after 48 hours of incubation at 28°C.

MFN1032 has been isolated from the sputum of a patient suffering from pneumonia. This bacterium has been characterized as *P. fluorescens biovar *I by polyphasic taxonomy, partial 16sRNA sequencing and siderotyping [7]. Culturability studies demonstrated that this strain was able to grow (Figure 1A) and formed biofilms on polystyrene at 37°C. As PAO1, this bacterium shown strong beta-hemolytic activity on sheep, horse or rabbit blood-agar plates (Figure 1B), whereas strain MF37 was non-hemolytic. MFN1032 also displayed lecithinase activity on egg-yolk agar plates (Figure 1C).

### Cytotoxic effects of bacterial culture supernatants

We assessed the cytotoxicity of supernatants from *P. fluorescens *MFN1032, *P. fluorescens *MF37 and *P. aeruginosa *PAO1 in two experimental models: LDH release from rat glial cells and the hemolysis of sheep erythrocytes in a liquid assay.

**Figure 2:**
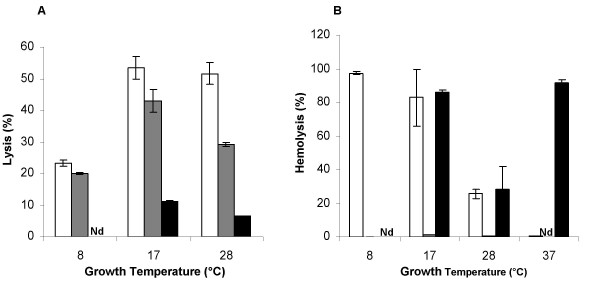
**Effect of growth temperature on the cytotoxicity of MFN1032 (white square), MF37 (grey square), and PAO1 (black square)**. After 15 generations in LB medium, aliquots of cell-free supernatants were assayed for glial cell lysis (A) or hemolysis (B), as described in the materials and methods. For assays of glial cell cytotoxicity, supernatants were concentrated with an Amicon ultra-15 filter, and then resuspended in glial cell medium. Each experiment was performed at least three times in triplicate. Nd: not determined.

We first investigated cytotoxicity in rat glial cells in primary culture, a model previously used to assess the cytotoxicity of *P. aeruginosa *and *P. fluorescens *[10]. Cell-free supernatants from stationary growth phase cultures were incubated overnight with primary cultures of rat glial cells. MFN1032 and MF37 supernatants were much more cytotoxic than those from the opportunistic pathogen PAO1 at all growth temperatures studied (Figure 2A). MFN1032 supernatants from bacteria grown at 17°C and 28°C were equally toxic (55% lysis), whereas those from bacteria grown at 8°C were loss toxic (25% lysis). MF37 also displayed significantly higher levels of secreted cytotoxic activity against glial cells than PAO1 grown at 17°C or 28°C. This activity was clearly temperature-dependent, and was maximal at 17°C.

We monitored the levels of secreted hemolytic activity displayed by MFN1032 throughout bacterial growth. MFN1032 grown at 17°C displayed hemolytic activity only at the start of the stationary phase and similar profiles were observed at 8°C and 28°C. The kinetics of hemolysin production by PAO1 was investigated only at 37°C. Hemolytic activity appeared to be stable after 15 bacterial generations in culture, in all the cases. These conditions were therefore adopted in all subsequent studies. Major differences were observed in the hemolytic activities of supernatants from cultures of MFN1032, MF37 and PAO1 grown at 8, 17, 28 and 37°C (Figure 2B). Culture supernatants from MF37 grown at 8, 17 and 28°C were non hemolytic (no test was carried out at 37°C because this strain cannot grow at this temperature). Hemolysis levels for the clinical strain MFN1032 were maximal at growth temperatures of 8 and 17°C (100% and 83% lysis, respectively), and lower at 28°C (26% lysis). MFN1032 supernatants were non hemolytic if the bacteria were cultured at 37°C. MFN1032 and PAO1 supernatants had similar hemolytical potentials if the bacteria were grown at 17°C (90% hemolysis) or 28°C (25% hemolysis) (Figure 2B). The hemolytic activity of PAO1 supernatants was maximal at growth temperatures of 37°C (optimal growth rate) and 17°C. By contrast, the secreted hemolytic activity of the MFN1032 strain was not maximal at the optimal growth rate for this strain (30°C).

As the *P. fluorescens *clinical strain MFN1032 displayed hemolysis, we investigated the factors potentially involved in this hemolytic activity.

### Measurement of protease activity, LPS and biosurfactant release and lecithinase activity in the supernatant

No protease activity or LPS release (KDO level) into the supernatants of MF37, MFN1032 and PAO1 cultured in LB medium at 28°C was detected (Table 1). We used egg yolk as a substrate for estimating PLC activity and biosurfactant release was assessed with the drop-collapse test. Both lecithinase activity and biosurfactant were detected in these three culture supernatants, although higher levels were observed for MFN1032 and PAO1 than for MF37 (Table 1).

**Table 1:** Release of protease, LPS, lecithinase and biosurfactant into LB supernatants after growth at 28°C: (+: detection, -: no detection).

**Strain**	**Protease**	**KDO**	**Lecithinase**	**BS**
MFN1032	-	-	++	++
MF37	-	-	+	+
PAO1	-	-	++	++

### Effect of D609 on the secreted hemolytic activity of MFN1032

We investigated the possible involvement of a secreted PLC in the hemolytic activity of MFN1032 using D609, which specifically inhibits certain PLCs [25]. Supernatants from MFN1032 were incubated at room temperature for 1 h with D609 at a final concentration of 1 mM, and their hemolytic activity was then assessed. Incubation with D609 decreased hemolytic activity by 68% (Table 2). Thus, a phospholipase C sensitive to D609 is clearly involved in the hemolytic activity of MFN1032. By contrast, D609 treatment did not decrease the hemolytic activity of PAO1 supernatants, consistent with previous results [26].

**Table 2:** Effect of D609 on the secreted hemolytic activities of MFN1032 and PAO1

	**% Hemolysis**	
	
**D609**	**-**	**+**
**Strain**		
MFN1032	92 ± 1	30 ± 3
PAO1	86 ± 1	85 ± 2

### Structural characterization of the PLC from MFN1032

**Figure 3:**
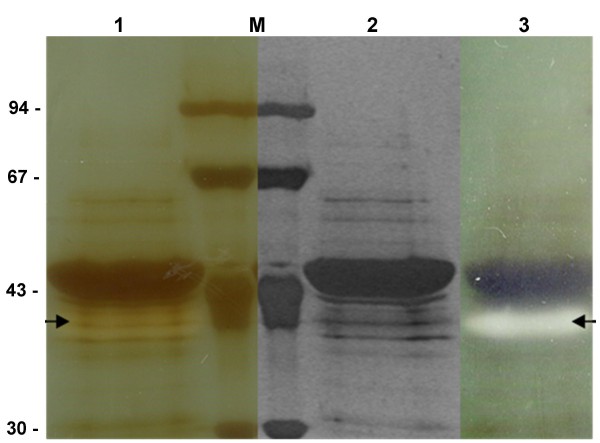
**Zymogram on egg-yolk agar of a silver-stained SDS-PAGE gel of MFN1032 supernatant**. PLC activity was detected as an opaque band on the plate (arrow), the molecular mass of which was deduced. Concentrated MFN1032 supernatant was obtained following growth at 17°C in LB medium. **1**: Front of a silver-stained gel placed on an egg-yolk agar plate (zymogram); **2**: corresponding silver-stained gel; **3**: other side of the zymogram; **M**: molecular size markers.

SDS-PAGE and zymograms were used to determine the apparent molecular mass of an enzyme with lecithinase activity. A single band with lecithinase activity was recovered from a silver-stained SDS-PAGE gel placed on an egg-yolk agar plate (Figure 3). This protein had a molecular mass of approximately 42 kDa. We designed PCR primers based on the gene encoding a PLC of similar molecular mass described by Preuss *et al*. and produced by an uncharacterized *P. fluorescens *strain [15]. A single fragment of 1.2 kb was obtained and cloned with the pMOS kit. This fragment was sequenced by Genome Express (France) and the sequence registered in the Genbank database (accession number: DQ462712) named *plcC*. This sequence encodes a predicted PlcC protein of 385 residues, with a molecular mass of 42 kDa. These findings are consistent with SDS-PAGE and suggest that the protein is secreted without cleavage. *In silico *analysis indicated the absence of any known secretion signal sequence. No gene amplification was obtained if the same primers were used with MF37 and PAO1 (data not shown).

A search of the NCBI nucleotide and protein database showed that the PLC from MFN1032 (PlcC) was very similar to putative PLCs from other strains of *P. fluorescens *(Table 3). The molecular mass of this group of PLCs ranges from 42 kDa to 59 kDa. PlcC is clearly related to the PLC described by Preuss *et al*. (98% identity), which was produced by a *P. fluorescens *strain isolated as a contaminant of placental extract of unknown origin. These two PLCs are also very similar to a putative PLC encoded by the *P. fluorescens *SBW25 genome (92% identity), but are more distantly related to the putative PLCs identified for the Pf-5 strain – a rhizosphere strain of *P. fluorescens *(76% identity) – and Pf0-1 (65% identity). Sequence identity was also observed with putative PLCs from the entomopathogen *Pseudomonas entomophila *(68%) and from *Legionella pneumophila*, although the level of identity was lowers (43%). No significant similarity was observed with the PLCs produced by *P. aeruginosa *(PlcH, PlcN and PlcB) or with the PC-PLC from *B. cereus *(Table 3).

**Table 3:** Comparison of the sequence of the MFN1032 PlcC with other PLC sequences

**Strain**	**PLC (ID Protein number)**	**% Identity to PlcC**	**Amino-acid residues**	**MW (kDa)**
*P. fluorescens *MFN1032	PlcC (GI 92090692)	-	385	42
*P. fluorescens *(Preuss)	- (GI 11611250)	98%	385	42
*P. fluorescens *SBW25	Putative	92%	383	42
*P. fluorescens *Pf-5	Putative (GI 68342549)	76%	546	59
*P. entomophila*	Putative (GI 104780281)	68%	544	60
*P. fluorescens *Pf0-1	Putative (GI 77380231)	65%	437	48
*L. pneumophila*	PlcA (GI 52627367)	43%	423	48
*P. aeruginosa*	PlcH (GI 15596041)	NS	730	79
*P. aeruginosa*	PlcN (GI 9949449)	NS	692	78
*P. aeruginosa*	PlcB (GI 9945846)	NS	328	37
*B. cereus*	PC-PLC (GI 28414376)	NS	283	28,5

NS: not significant

### Construction of MFN1032 plcC mutants

The MFN1036 strain, a clone of MFN1032 that overexpresses *plcC*, was obtained by electroporating MFN1032 with pUCP20, which carries the *plcC *gene and its promoter (1.5 kb). The expression of *plcC *from this plasmid was under the control of the constitutive *plac *promoter. We investigated the involvement of PlcC in hemolytic activity by culturing MFN1036 at 17°C and 28°C and assessing its hemolytic activity in liquid assays. MFN1036 was significantly more cytotoxic (74% lysis at 28°C) than the parental strain MFN1032 (26% lysis at 28°C) (Table 4). We used a *plcC*-deficient mutant, MFN1037, to determine whether PlcC contributed to the hemolytic activity of MFN1032. We detected no hemolysis with MFN1037 culture supernatants, regardless of growth temperature (Table 4). Complementation of the *plcC *mutation with pMF36 (strain MFN1038) did not restore the hemolytic phenotype of MFN1032 (Table 4), whereas it did result in levels of lecithin degradation on egg-yolk agar plates similar to those for the wild-type strain.

**Table 4:** Comparison of the secreted hemolytic activities of MFN1032 and the *plcC *mutants at various growth temperatures

**Growth Temperature (°C)**	8	17	28
**Strain**	**% Lysis**		

MFN1032	98 ± 1	83 ± 17	26 ± 3
MFN1036	Nd	100 ± 2	74 ± 13
MFN1037	2 ± 1	0.5 ± 0.1	0.8 ± 0.2
MFN1038	2 ± 1	0.4 ± 0.1	1 ± 0.2

**Figure 4:**
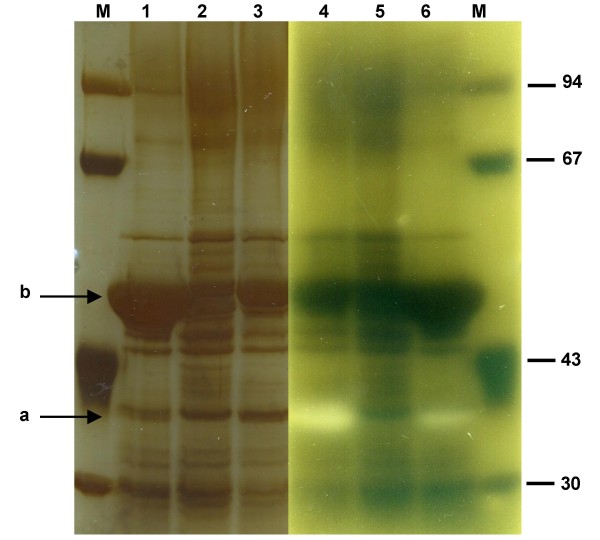
**SDS-PAGE of total extracellular proteins of MFN032 and the PlcC mutants MFN1037 and MFN1038**. Supernatants of cultures at 17°C in LB medium were concentrated on an Amicon ultra-15 filter and subjected to SDS-PAGE in a 10% acrylamide gel. The gel was silver-stained (lanes 1 to 3) and placed against an egg-yolk agar plate (lanes 4 to 6) to reveal lecithinase activity (the opaque band on the plate). **1 **and **6**: MFN1032; **2 **and **5**: MFN1037 (*plcC*-deficient MFN1032); **3 **and **4**: MFN1038 (*plcC *complemented MFN1037). **M**- molecular size markers. **a**: PlcC; **b**: flagellin.

SDS-PAGE analysis of total extracellular proteins followed by PlcC zymogram detection on egg-yolk agar plates showed that there was no PlcC in the supernatant of cultures of the *plcC*-deficient mutant MFN1037, as no lecithin degradation was observed (Figure 4, lane 2 and 5, arrow a). The mutant released smaller amounts of an approximately 50 kDa protein than did MFN1032 (arrow b). Analysis of the amino-acid sequence of the N-terminus of this protein (ALTVNTNIAS) suggested that it was a homolog of the flagellin protein FliC produced by *P. aeruginosa *and *P. putida*. The *plcC*-complemented strain MFN1038 displayed wild-type levels of lecithin degradation in zymogram analysis and a partial restoration of flagellin levels (Figure 4, lanes 3 and 4, bands a and b respectively). MFN1036 had an electrophoretic profile identical to that of MFN1032, except for the 42 kDa protein previously identified as PlcC, which was produced in larger amounts by MFN1036 (data not shown).

### Pleiotropic effects of *plcC *gene mutation

**Figure 5:**
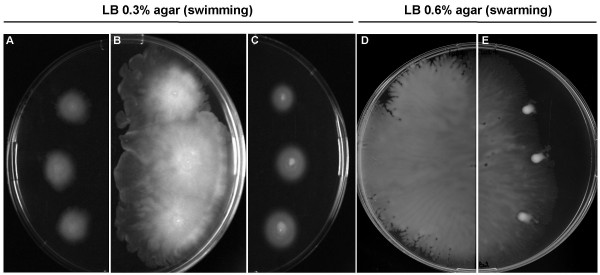
**Motility assays for MFN1032, MFN1036 and MFN1037**. The swimming motility of MFN1032 (A), MFN1036 (B) and MFN1037 (C) on 0.3% LB agar plates and the swarming motility of MFN1032 (D) and MFN1037 (E) on 0.6% LB agar plates after 16 h of incubation at 28°C. MFN1032 (wild type) and MFN1037 (*plcC*-deficient MFN1032) presented concentric halos on 0.3% agar, corresponding to swimming motility, wherease MFN1036 (*plcC*-overexpressing MFN1032) displayed a dendritic pattern indicative of swarming motility. The same pattern of MFN1037 mobility was obtained with strain MFN1038 (data not shown).

Flagellin levels in the supernatant of the *plcC*-deficient strain MFN1037 were low. We therefore investigated the motility of the MFN1032 and the *plcC *mutants. In swimming conditions (i.e 0.3% agar, final concentration), MFN1036 completely invaded the plate over the course of a 16 h incubation at 28°C and displayed swarming motility, whereas MFN1032 displayed only swimming motility and diffused less (Figure 5B and 5A, respectively). This may be due to higher levels of biosurfactant release or production, than observed for the wild-type strain, as suggested by the translucent appearance of the halo on the plates. This hypothesis was confirmed by the drop-collapse test (Table 5). The *plcC*-deficient mutant MFN1037 and the complemented strain MFN1038 (not shown) had swimming motility patterns similar to those of MFN1032 (Figure 5C), but did not produce biosurfactant on LB agar plates, as shown by the drop-collapse test (Table 5). In motility assays on LB plates containing 0.6% agar, MFN1037 and MFN1038 had no swarming motility and no movement was observed even after 40 h of growth at 28°C, whereas MFN1032 continued to be motile (Figure 5D and 5C, respectively).

**Table 5:** Evaluation of biosurfactant release into culture supernatants by the drop-collapse test

**Strain**	**BS Production**
MFN1032	++
MFN1036	+++
MFN1037	-
MFN1038	-

+: detection; -: no detection

**Figure 6:**
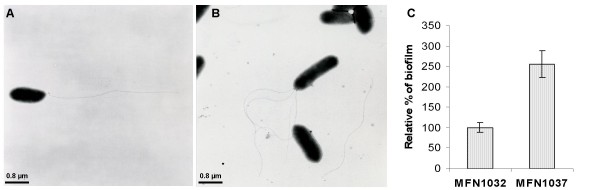
**Transmission electron microscopy (TEM) of MFN1032 (A) and MFN1037 (B) cells. (C) Biofilm quantification of MFN1032 and MFN1037 after 24 h of growth at 37°C in a polystyrene microtiter plate**. Biofilm formation was quantified as the percentage relative to that observed with MFN1032 grown at 37°C.

The loss of biosurfactant production may be enough, in itself, to account for the loss of swarming motility of the *plcC*-deficient strain, but cannot account for the low levels of flagellin release. Electron microscopy analyses of MFN1032 and MFN1037 cells showed that the wild-type strain had a single polar flagellum whereas the *plcC*-deficient strain MFN1037 displayed a hyperflagellated phenotype, with most of the cells presenting three polar flagella (Figure 6A and 6B, respectively). No difference in flagellum size was observed, but these differences in flagellation would probably affect the properties of the two strains. MFN1037 cells formed aggregates on the grid, whereas MFN1032 cells did not, despite having been prepared in the same conditions. Adhesion was evaluated by analyzing biofilm formation on polystyrene microarrays at 37°C. We found that MFN1037 had a greater capacity to form biofilms than the wild-type strain (255 ± 32% more adhesion than MFN1032 at 37°C; Figure 6C).

### Analysis of the flanking regions of *plcC*

**Figure 7:**
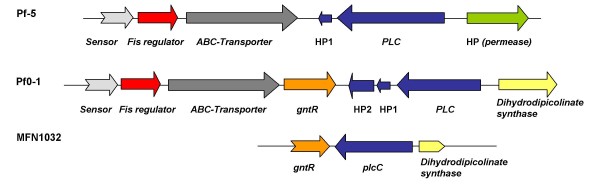
**Genomic organization and flanking regions of the *plcC *gene from MFN1032 and the putative phospholipase C genes in *P. fluorescens *Pf0-1 and Pf5**. HP: hypothetical protein

We investigated the genomic organization of the genes encoding PLCs in the genomes of *P. fluorescens *Pf0-1 and Pf5. We identified two genes of unknown function in the operon containing the *plc *gene in Pf0-1, and one in Pf5 (Figure 7). We found no other gene in an operon with *plcC *in MFN1032, confirming that the pleiotropic effects of *plcC *disruption could not be due to a polar mutation. No gene encoding a potential flagellin or belonging to the flagellar regulon were found in the surrounding region. A gene encoding a putative dihydropycolinate synthase was found in the 5' flanking region of *plcC*, in an antisense orientation. In the 3' region, a similar phenomenon was observed, except that the gene encoded a putative transcriptional regulator of the GntR family [27]. This regulator is probably involved in *plcC *expression because the consensus GntR binding site (T.GT-N(0-3)-AC.A), as identified by Rigali *et al*. [27], is present in the sequence immediately upstream from the *plcC *gene.

## Discussion

*P. fluorescens *is generally considered to be non pathogenic, but its infectious potential has nonetheless been demonstrated. *In vitro *studies of environmental strains, including *P. fluorescens *MF37, have shown that this psychrotrophic bacterium has most of the features of an opportunistic pathogen [8]. *P. fluorescens *is a highly heterogeneous species, ranging from avirulent strains that can be used in crop protection [28] to clinical strains involved in lung, urinary tract and blood infections. However, whereas the low virulence of industrial strains has been studied in detail, the factors involved in the virulence of clinical forms of *P. fluorescens*, including MFN1032, have not previously been investigated. Virulence results from the combined effects of direct contact between the bacterium and the target cell and the production of many soluble or secreted molecules and exotoxins acting at some distance from the microorganism. MFN1032 has been shown to be highly cytotoxic *in vitro *to eukaryotic cells, to which it binds [7]. We investigated the role of factors secreted by this bacterium in cytotoxicity. We therefore carried out experiments with supernatants and culture medium extracts only.

In addition to its ability to multiply at 37°C, which is itself unusual for a strain of *P. fluorescens*, MFN1032 generated molecules with high levels of hemolytic and cytotoxic activity, as observed in *in vitro *models. The hemolytic activity of MFN1032 supernatants was similar to that of *P. aeruginosa*, which is an opportunistic pathogen. By contrast, a typical psychrotrophic strain, MF37, displayed no hemolytic activity under the same conditions. More surprisingly, MFN1032 supernatants were significantly more cytotoxic to rat glial cells than PAO1 supernatants, for bacteria grown at 17°C and 28°C. The secreted hemolytic and cytotoxic activities of *P. fluorescens *MFN1032 and *P. aeruginosa *PAO1 appeared to be temperature-dependent, as was *P. fluorescens *MF37 cytotoxicity. Surprisingly, the supernatant of MFN1032 grown at 37°C displayed no hemolysis. At first glance, this finding appears to contradict the hypothesis that hemolysis is a virulence factor. Low temperature induction likely reflects the evolutionary history of the factors involved in this activity and the function of those elements in non-infective conditions. In fact, it has been described that *Pseudomonas aeruginosa *from clinical and non-clinical environments are genetically and functionally similar [29,30]. So evolution of virulence determinants in opportunistic pathogens is not necessary linked to their interaction with the human host. Finally, invasin and the heat-stable enterotoxin Yst from *Y. enterocolitica *are also virulence factors produced preferentially at temperatures below 37°C *in vitro*. However, in conditions of slight acidity or high osmolarity, these virulence genes are expressed at 37°C *in vitro*. High-temperature repression may therefore be overcome by other conditions stimulating expression in the host.

We found that neither protease nor LPS was responsible for the extracellular hemolytic activity of MFN1032. The absence of protease activity in these conditions was not surprising, as protease activity has been recovered from *P. aeruginosa *PAO1 only after at least 18 h of growth in LB medium and has never been observed with *P. fluorescens *MF0 in this medium [13]. Many pathogens display secreted hemolytic activity, which may be due to toxins, biosurfactants, and/or enzymes (essentially PLCs) [31]. Various species of *Pseudomonas *have been reported to produce hemolytic and/or non hemolytic PLCs [16,21,32]. The observed degradation of lecithin by MF37 on plates or in supernatants is consistent with this bacterium producing a non hemolytic PLC other than PlcC, as no gene amplification was observed with *plcC *primers. By contrast, the PLC produced by MFN1032 is involved in the hemolytic activity of this strain, as shown by the inhibitory effect of D609.

A protein with lecithinase activity was identified in MFN1032 supernatants by an egg-yolk agar plate zymogram method. This enzyme has a molecular mass of 42 kDa, as determined by SDS-PAGE. This apparent molecular mass is similar to that of previously described *P. fluorescens *PLC enzymes [15,16,18] and those of the putative PlcB [33] and PlcA produced by *P. aeruginosa *(Vasil *et al*., data communicated at the *Pseudomonas *Congress, 2005). The PlcH and PlcN produced by *P. aeruginosa *have a higher molecular mass [23].

Analysis of the sequence of the *plcC *gene indicated considerable similarity between PlcC and putative PLCs identified in various strains of *P. fluorescens *including, in particular, the PLC produced by a *P. fluorescens *isolated from placental extract [15] and the putative PLC from SBW25. This *plcC *gene was shorter than those predicted for Pf0-1 and Pf5, and the main difference between the protein encoded by this gene and Pf0-1 and Pf5 concerned the N-terminus of the protein. It is unclear whether *plc *genes are expressed in the other strains and it is possible that PLC production is not specific to adaptation to human infection but was acquired to survive in other environments.

No significant sequence similarity was found between the group of PLCs described here and the well characterized zinc-metallophospholipases C of Gram-positive bacteria. This group of PLCs is also only very distantly related to the enzymes described by Stonehouse *et al*., including the PlcH and PlcN of *P. aeruginosa *[26]. The differences between these enzymes concern not only their sequences, but also their catalytic sites, as D609 has no effect on PlcH activity [26]. The PlcC from MFN1032 also appears to be different from PAO1 PlcB and PlcA [33]. Thus, although *P. fluorescens *and *P. aeruginosa *are generally thought to be closely related, they have different PLCs. Preuss *et al*. reported an elegant, detailed biochemical characterization of their PLC. Their data concerning iron requirement and D609 susceptibility suggested the existence of a new class of PLCs, to which MFN1032 PlcC might belong.

Neither PlcC nor the PLC studied by Preuss *et al*. has a signal peptide. These enzymes are therefore presumably secreted by an unknown mechanism, whereas most PLCs are secreted by the Tat or Sec pathways [33-35]. Several phospholipases have been reported to belong to the flagellar regulon [36] and to be secreted by the flagellar export apparatus [37,38], a type-three secretion system [39], or by the two-partner secretion (TPS) system [40]. For example, the transcription and secretion of YplA, the phospholipase A_1 _of *Yersinia enterocolitica*, are controlled by the flagellar regulon [37]. Warren *et al*. suggested that an amino-terminal secretion signal of about 20 amino acids is required for YplA secretion, but did not identify a particular signal peptide motif [41]. Some PLCs have been shown to be regulated by the flagellar regulon, but no evidence has ever been published to suggest that PLCs may affect the expression of this regulon. There is no evidence to suggest that the PlcC of MFN1032 is secreted by the flagellar apparatus, but the lower levels of flagellin in the supernatant of the *plcC*-deficient mutant than in the supernatant of the control or complemented strain implies a close link between these two proteins. This hypothesis is supported by *in silico *analysis, which identified a cluster of orthologous groups (COG) corresponding to a flagellar hook motif in PlcC. It should be noted that the lower levels of flagellin do not indicate the lack of flagella. The mutant displayed swimming motility and TEM observations of MFN1037 cells confirmed that flagella were present.

We constructed a *plcC*-overexpressing mutant of MFN1032, MFN1036, to investigate the involvement of PlcC in hemolytic activity and we found that a higher level of PlcC production by MFN1032 was associated with higher levels of hemolytic activity. The loss of hemolytic activity in the *plcC*-deficient strain MFN1037 confirmed the involvement of this enzyme in hemolysis. Hemolytic activity was not restored in the MFN1038 strain, which overexpressed *plcC*, and extinction of the *plcC *gene had also pleiotropic effects, particularly as concerns the release of biosurfactant and flagellin. This was not due to a polar mutation involving *plcC *gene interruption, as the *plcC *gene was not associated with any other gene in an operon in the MFN1032 genome. Linares *et al*. described that the presence of low antibiotics concentrations in the culture media may induce changes in bacterial physiology (biofilm, motility and cytotoxicity) [42]. Their data could explain the phenotype change of the *plcC *mutant MFN1037, but we were not in conditions used by these authors (i.e subinhibitory concentrations of antibiotics). We probably disturb a complex regulatory network in MFN1037, and this hypothesis is also supported by *plcC *overexpression in MFN1036 resulting in the overproduction of biosurfactants, increasing swarming mobility.

Such complex regulatory systems often involve positive and/or negative feedback loops [43]. GntR regulators have been reported to maintain their own expression [44], so a positive feedback loop regulating GntR levels may exist. As previously reported, a simple change affecting a key element of this kind of system might lead to epigenetic modification [45]. Epigenetic switches, corresponding to phenotype modifications, arise and may be transmitted from a cell to its progeny in the absence of genetic modifications. This type of regulation has been reported for the cytotoxicity associated with the T3SS (type-three secretion system) of *P. aeruginosa*. Transient expression of the ExsA transcriptional regulator in non inducible strains leads to the acquisition of a cytotoxic phenotype [46]. The artificial extinction of *plcC *in MFN1037 may have triggered such an epigenetic switch.

## Conclusion

These findings demonstrate that some *P. fluorescens *strains have some of the key characteristics of opportunistic pathogens. We provide the first demonstration, to our knowledge, of the involvement of a PLC in the secreted hemolytic activity of a clinical strain of *P. fluorescens *(MFN1032). We found that MFN1032 secretes a phospholipase C homologous to a PLC from an uncharacterized *P. fluorescens *strain previously studied biochemically by Preuss. This enzyme belongs to a new group unrelated to the PLCs produced by *P. aeruginosa *and seems to be produced by a wide range of *P. fluorescens *strains, although no homolog of the *plcC *gene was found in our model strain, MF37. Further studies including strains of different origins presenting hemolytic activity would clarify these observations. However, although PlcC is not specific to clinical isolates, this enzyme is a potential virulence factor as our data show that this enzyme is directly involved in the secreted hemolytic activity of MFN1032, as demonstrated by the inhibitory effect of D609. The direct involvement of PlcC in MFN1032 virulence could be further demonstrated or excluded by studying in *vivo *models.

Results obtained with the *plcC *mutants also suggest that this enzyme interferes with biosurfactant production, which might also account for the higher levels of hemolysis observed when *plcC *was overexpressed. The pleiotropic phenotype resulting from *plcC *mutation or overexpression suggests that PlcC is involved in a regulatory network. We are now investigating a possible role for the 3' flanking region of *plcC*, corresponding to the putative transcriptional regulator GntR, with the aim of determining the link between PlcC, GntR and biosurfactant production.

## Methods

### Bacterial strains and culture conditions

The MFN1032 strain was collected from a patient suffering from pulmonary tract infection (expectoration) at a hospital in *Haute Normandie*. This strain was the only bacterial contaminant in a normally sterile compartment and was considered to be the cause of the infection. MFN1032 was identified as a *P. fluorescens *biovar I strain [7]. PAO1 is a *P. aeruginosa *strain widely used in laboratory studies [47]. MF37 is a spontaneously rifampicin-resistant mutant of the MF0 strain, a psychrotrophic strain of *P. fluorescens *isolated from unpasteurized milk extensively studied in our laboratory [48]. These bacteria were cultured in Luria Bertani medium (LB), at various temperatures between 8 and 37°C, with shaking at 180 rpm. When necessary, 500 μg/mL mezlocillin or 40 μg/mL tetracycline was added. Bacterial density was determined by measuring optical density at 580 nm (Spectronic 601 spectrophotometer).

### Glial cell cytotoxicity assays

Cytotoxicity was assessed by quantifying lactate dehydrogenase (LDH) release into the medium by cells, as this release reflects levels of necrosis. Concentrated supernatants (10 μL/mL culture medium) from bacterial cultures at various temperatures were incubated overnight with rat glial cells (8 × 10^6 ^cells/mL) that had been cultured *in vitro *for 12 days. Controls included LB concentrated with Amicon Ultra-15 centrifugal filter units and incubated for the same period of time in culture medium for glial cells. The Cytotox 96^® ^enzymatic assay (Promega, France) was used to quantify necrosis by measuring LDH release into the culture medium. The percentage total lysis was calculated as follows: % = [(X-B)/(T-B)] × 100, where B (baseline) is a control corresponding to LDH spontaneously produced by glial cells incubated with concentrated LB (10 μL/mL culture medium), T is a positive control (100%) corresponding to the amount of LDH detected in the culture medium after total lysis of the cell population by Triton X-100 (9% (v/v) in water) treatment and X is the amount of LDH detected in the culture medium of the sample tested. The assay was sensitive enough to measure LDH concentrations equivalent to the lysis of 1% of the cell population.

### Hemolysis assays

The hemolytic potentials of bacterial culture supernatants were measured by a technique derived from that described by Dacheux *et al*. [49]. Briefly, sheep erythrocytes obtained from Eurobio (France) were washed three times in PBS (pH 7.2, 0.8% NaCl, 0.02% KCl, 0.17% Na_2_HPO_4_, 0.8% KH_2_PO_4_) and resuspended in RPMI-1640 medium (Sigma) to a final concentration of 2% (cell volume/medium volume). Bacteria were grown in LB, and the enzymatic activity of the culture supernatant was assessed. Samples were obtained from bacteria cultured at various temperatures (8, 17, 28 or 37°C) for 15 generations. The bacterial cultures were centrifuged and the supernatants collected and sterilized by passage through a Millipore filter with 0.22 μm pores. For hemolysis assays, we combined 600 μL of a 2% suspension of red blood cells (RBCs) with 600 μL of filtered supernatant and incubated this mixture for 2 h at 37°C. The suspension was centrifuged at 10,000 g for 8 min at 4°C, and hemoglobin release was assessed by determining absorbance at 540 nm. The percentage (%) of cells lysed was calculated as follows: % = [(X-B)/(T-B)] × 100. B (baseline) is a negative control corresponding to RBCs incubated with 600 μL sterile LB and T is a positive control corresponding to the total lysis obtained by incubating RBCs in LB supplemented with 0.1% SDS (final concentration). X is the absorbance value for the sample analyzed. All experiments were performed at least three times in triplicate. D609 was obtained from Sigma and used at a final concentration of 1 mM.

### Protease and LPS assays

Protease assays were carried out with supernatants from bacteria cultured for 15 generations, as described by Hellio *et al*. [13]. LPS levels were quantified by determining 2-keto-3-deoxyoctulonic acid (KDO) concentration, as described by Karkhanis *et al*.[50].

### Biosurfactant production

Biosurfactant production was assessed by the drop-collapse test mainly as described previously [51]. Drops of Volvic water were dispensed into a Petri dish with a polystyrene platform. Drops of culture supernatant were added to the drops of water. If the culture broth contained biosurfactant, the droplets of water collapsed.

### Gel electrophoresis conditions, zymogram methods and amino-acid sequencing

Sodium dodecyl sulfate polyacrylamide gel electrophoresis (SDS-PAGE) was carried out as described by Laemmli [52]. For a zymogram on egg-yolk agar, a silver-stained SDS-PAGE gel was washed three times in water and placed directly on an egg-yolk agar plate. After overnight incubation at 37°C, lecithinase activity was detected as an opaque band corresponding to lecithin hydrolysis. For N-terminal amino-acid sequencing, the supernatant proteins were transferred to PVDF membranes and subjected to Edman degradation in an Applied Biosytems 492 automated protein sequencer.

### Motility assays

Motility assays were performed as described [53], with light modifications. Each strain was incubated on LB agar plates for 24 h at 28°C. Plates of LB medium solidified with 0.3% agar (for the assessment of swimming motility) were inoculated by stabbing colonies with a toothpick and inserting the end of the toothpick just below the surface of the agar. Three colonies were picked from three plates and incubated at 28°C until a migration halo appeared. We then spotted 5 μL of 3 independent suspensions of each strain onto LB medium plus 0.6% agar (swarming motility) and the plates were incubated until a migration halo appeared.

### Static biofilm assay and quantification

Biofilm assay was adapted from the method of O'Toole and Kolter [54]. Bacteria were plated on LB agar plates and incubated for 24 h at 28°C. Three independent LB suspensions of each strain were adjusted to an OD_580 _of 0.4. We added 100 μL aliquots of each suspension to the wells of 96-well microtiter plates, at least in triplicate. The negative control consisted of LB broth without bacteria. Plates were incubated for 24 h at 37°C without shaking. The bacterial cells bound to the wall of the wells were then stained with 0.1% crystal violet and the remaining crystal violet was then quantified by treatment with 150 μL of 1% SDS and the determination of absorbance at 595 nm with a microtiter plate reader (Model 680XR, Biorad).

### Electron microscopy

Early exponential growth phase bacteria were fixed by incubation in an equal volume of modified Karnofsky buffer (2.5% glutaraldehyde, 1% paraformaldehyde in 0.15 M sodium cacodylate buffer) at least 10 minutes and washed in phosphate buffer (0.1 M; pH 8). Nickel-coated copper grids (200 mesh) were floated on a drop of washed bacteria, rinsed in ultrapure grade water, and negatively stained with 0.5% (wt/vol) phosphotungstic acid (5 to 10 s). Electron microscopy was performed with a Philips CM10 transmission electron microscope.

### Oligonucleotides and polymerase chain reactions

Five colonies of each strain were suspended in 600 μL sterile ultrapure water. The suspension (1 μL) was then used for PCR with template DNA from bacterial colonies. PCR was carried out in a 50 μL reaction volume, in a GeneAmp PCR system 2400 (Perkin-Elmer Corporation, USA). Each reaction mixture contained DNA template, 1.25 μL *Taq *polymerase (GMP grade, Roche Diagnostics Gmbh, Germany),*Taq *PCR buffer (GMP grade, Roche Diagnostics Gmbh, Germany), 0.2 μL primers and 125 μM of each deoxyribonucleoside triphosphate. After initial denaturation for five minutes at 94°C, the reaction mixture was subjected to 30 cycles of 1 minute at 94°C, 30 s at 54°C and 1.5 minutes at 72°C, followed by a final 7-minute extension at 72°C. The primers used for PCR were purchased from Proligo (France). The complete *plcC *gene sequence (approximately 1.2 kb) was amplified with PLC1 (5'-ATGTCAGGTCTTGAACTCGCA-3') and PLC2 (5'-TTAGTTGGCGGGTTGGTTT-3'). The use of PLC0 (5'-GGTGGAAATCACCCTGG-3') with PLC2 amplified the *plcC *gene and its promoter (approximately 1.5 kb). GntR (5'-CCGAGTCGGCGATCATG-3') was used with PLC (5'-GCAAGGACGTCAACGATTTG-3') to amplify the 3' flanking region of *plcC*.

### Sequence determination and analysis

The 1.2 kb or 1.5 kb PCR fragment was isolated with a DNA gel extraction kit (Genomics/Millipore) and cloned with the pMOSBlue Blunt-ended Cloning Kit (Amersham/Biosciences). MOS cells were transformed and, after blue/white colony screening, clones were picked and plasmid DNA was isolated with the QIAprep Spin Miniprep Kit (Qiagen). Plasmids were checked by digestion with *Hin*dIII/*Ava*I and sequenced by Genome Express (France). The predicted protein sequence of MFN1032 PlcC was for BLAST queries .

### Construction of a *plcC*-overexpressing MFN1032 clone: MFN1036

The 1.5 kb PCR fragment was inserted into the pMOSBlue vector. The 1.5 kb *Ava*I/*Hin*dIII fragment was then transferred to the pUCP2O shuttle vector to construct pMF36 [55]. MFN1032 was electroporated with pMF36. Positive colonies were selected based on mezlocillin resistance and lecithin degradation on an egg-yolk agar plate. Clones were checked by plasmid DNA isolation with the QIAprep Spin Miniprep Kit (Qiagen), followed by enzymatic digestion. The strain obtained, MFN1036, was cultured in LB with a final concentration of 500 μg/mL mezlocillin.

### Construction of a *plcC*-negative mutant, MFN1037, and the complemented MFN1038 strain

*Eco*RI/*Hin*dIII digestion of the 1.2 kb PCR fragment generated a 1 kb fragment corresponding to a 3'-deleted plcC gene. This fragment was inserted into the transferable suicide plasmid pME3087 [56], creating pMF1034 in *Escherichia coli *DH5αMCR cells. Plasmids were isolated with the QIAprep Spin Miniprep Kit (Qiagen), checked by digestion with *Hin*dIII/*Eco*RI and transferred into the *E. coli *S17.1 conjugative strain [57]. MFN1032 cells were conjugated with the S17.1 pMF1034 strain and selected for resistance to tetracycline and ampicillin. Clones were tested by PCR with PLC0 and PLC2 probes to confirm disruption of the *plcC *gene. MFN1037 (*plcC*-deficient MFN1032) had no1.5 kb fragment corresponding to the *plcC *gene under the control of its own promoter. This strain also had an attenuated egg-yolk degradation phenotype. It was cultured in LB with a final concentration of 40 μg/mL tetracycline. Complementation of the mutation was obtained by electroporating MFN1037 with pMF36 (strain MFN1038). Clones were selected for resistance to mezlocillin and tetracycline and egg-yolk degradation phenotype, and were checked by plasmid DNA isolation with the QIAprep Spin miniprep Kit (Qiagen), followed by enzymatic digestion.

## Authors' contributions

GR wrote the manuscript and analyzed most of the data. AM participated in the molecular genetic studies, carried out the sequence comparison and participated in the design of the study and the manuscript. JG was involved in the swimming motility assays and flagellin identification. WV and OL carried out the glial cell cytotoxicity assays. MF helped to design and write the manuscript. NO was involved in designing the study. All authors read and approved the final manuscript.

## References

[B1] Whyte A, Lafong C, Malone J, Golda B (1999). Contaminated lithium heparin bottles as a source of pseudobacteremia. J Hosp Infect.

[B2] Spiers AJ, Buckling A, Rainey PB (2000). The causes of *Pseudomonas *diversity. Microbiology.

[B3] Von Graevenitz A (1973). Clinical microbiology on unsual *Pseudomonas *species. Progress in Clinical Pathology.

[B4] Bernstein DI, Lummus ZL, Santilli G, Siskosky J, Bernstein IL (1995). Machine operator's lung. A hypersensitivity pneumonitis disorder associated with exposure to metalworking fluid aerosols. Chest.

[B5] Kaushal ML, Grover PS, Gupta ML (1998). Non-fermenters in urinary tract infection. J Assoc Physicians India.

[B6] Hsueh P, Teng L, Pan H, Chen Y, Sun C, Ho S, Luh K (1998). Outbreak of *Pseudomonas fluorescens *bacteremia among oncology patients. J Clin Microbiol.

[B7] Chapalain A, Rossignol G, Lesouhaitier O, Merieau A, Gruffaz C, Guerillon J, Meyer JM, Orange N, Feuilloley MGJ (2008). Comparative study of seven fluorescent pseudomonad clinical isolates. Can J Microbiol.

[B8] Picot L, Abdelmoula SM, Merieau A, Leroux P, Cazin L, Orange N, Feuilloley MG (2001). *Pseudomonas fluorescens *as a potential pathogen: adherence to nerve cells. Microbes Infect.

[B9] Feuilloley MGJ, Mezghani-Abdelmoula S, Picot L, Lesouhaitier O, Merieau A, Guerillon J, Orange N (2003). Involvement of Pseudomonas and related species in central nervous system infections. Recent Adv Dev Microbiol.

[B10] Picot L, Mezghani-Abdelmoula S, Chevalier S, Merieau A, Lesouhaitier O, Guerillon J, Cazin L, Orange N, Feuilloley MG (2004). Regulation of the cytotoxic effects of *Pseudomonas fluorescens *by growth temperature. Res Microbiol.

[B11] Lazdunski A (2003). *Pseudomonas aeruginosa*: a model of choice for the study of opportunistic pathogen. Ann Fr Anesth Reanim.

[B12] Mohr CD, Tomich M, Herfst CA (2001). Cellular aspects of *Burkholderia cepacia *infection. Microbes Infect.

[B13] Hellio FC, Orange N, Guespin-Michel JF (1993). Growth temperature controls the production of a single extracellular protease by *Pseudomonas fluorescens *MF0, in the presence of various inducers. Res Microbiol.

[B14] Dieckelmann M, Johnson LA, Beacham IR (1998). The diversity of lipases from psychrotrophic strains of *Pseudomonas*: a novel lipase from a highly lipolytic strain of *Pseudomonas fluorescens*. J Appl Microbiol.

[B15] Preuss I, Kaiser I, Gehring U (2001). Molecular characterization of a phosphatidylcholine-hydrolyzing phospholipase C. Eur J Biochem.

[B16] Ivanov A, Titball RW, Kostadinova S (1996). Characterisation of a phospholipase C produced by *Pseudomonas fluorescens*. New Microbiol.

[B17] Sacherer P, Defago G, Haas D (1994). Extracellular protease and phospholipase C are controlled by the global regulatory gene *gac *A in the biocontrol strain *Pseudomonas fluorescens *CHA0. FEMS Microbiol Lett.

[B18] Crevel I, U S, Carne A, Katan M (1994). Purification and properties of zinc-metallophospholipase C from *Pseudomonas fluorescens*. Eur J Biochem.

[B19] Doi O, Nojima S (1971). Phospholipase C from *Pseudomonas fluorescens*. Biochim Biophys Acta.

[B20] Songer JG (1997). Bacterial phospholipases and their role in virulence. Trends Microbiol.

[B21] Titball RW (1993). Bacterial phospholipases C. Microbiol Rev.

[B22] Schmiel DH, Miller VL (1999). Bacterial phospholipases and pathogenesis. Microbes Infect.

[B23] Ostroff RM, Vasil AI, Vasil ML (1990). Molecular comparison of a nonhemolytic and a hemolytic phospholipase C from *Pseudomonas aeruginosa*. J Bacteriol.

[B24] Montes L-R, Ibarguren M, Goni FM, Stonehouse MJ, Vasil MI, Alonso A (2007). Leakage-free membrane fusion induced by the hydrolytic activity of PlcHR2, a novel phospholipase C/sphingomyelinase from *Pseudomonas aeruginosa*. Biochim Biophys Acta.

[B25] Amtmann E (1996). The antiviral, antitumoural xanthate D609 is a competitive inhibitor of phosphatidylcholine-specific phospholipase C. Drugs Exp Clin Res.

[B26] Stonehouse MJ, Cota-Gomez A, Parker SK, Martin WE, Hankin JA, Murphy RC, Chen W, Lim KB, Hackett M, Vasil AI (2002). A novel class of microbial phosphocholine-specific phospholipases C. Mol Microbiol.

[B27] Rigali S, Derouaux A, Giannotta F, Dusart J (2002). Subdivision of the helix-turn-helix GntR family of bacterial regulators in the FadR, HutC, MocR, and YtrA subfamilies. J Biol Chem.

[B28] Compant S, Brion D, Jerzy N, Christophe C, Barka EA (2005). Use of Plant Growth-Promoting Bacteria for Biocontrol of Plant Diseases: Principles, Mechanisms of Action, and Future Prospects. Applied and Environmental Microbiology.

[B29] Alonso A, Rojo F, Martinez JL (1999). Environmental and clinical isolates of *Pseudomonas aeruginosa *show pathogenic and biodegradative properties irrespective of their origin. Environ Microbiol.

[B30] Morales G, Wiehlmann L, Gudowius P, van Delden C, Tummler B, Martinez JL, Rojo F (2004). Structure of *Pseudomonas aeruginosa *populations analyzed by single nucleotide polymorphism and pulse-field gel electrophoresis genotyping. J Bacteriol.

[B31] Alouf J (2000). Les toxines bactériennes membranolytiques. Bull Soc Fr Microbiol.

[B32] Sonoki S, Ikezawa H (1975). Studies on phospholipase C from *Pseudomonas aureofaciens*. I. Purification and some properties of phospholipase C. Biochim Biophys Acta.

[B33] Barker AP, Vasil AI, Filloux A, Ball G, Wilderman PJ, Vasil ML (2004). A novel extracellular phospholipase C of *Pseudomonas aeruginosa *is required for phospholipid chemotaxis. Mol Microbiol.

[B34] Voulhoux R, Ball G, Ize B, Vasil ML, Lazdunski A, Wu LF, Filloux A (2001). Involvement of the twin-arginine translocation system in protein secretion via the type II pathway. Embo J.

[B35] Rossier O, Cianciotto NP (2005). The *Legionella pneumophila tat*B gene facilitates secretion of phospholipase C, growth under iron-limiting conditions, and intracellular infection. Infect Immun.

[B36] Givskov M, Eberl L, Christiansen G, Benedik MJ, Molin S (1995). Induction of phospholipase- and flagellar synthesis in *Serratia liquefaciens *is controlled by expression of the flagellar master operon *flh*D. Mol Microbiol.

[B37] Schmiel DH, Young GM, Miller VL (2000). The *Yersinia enterocolitica *phospholipase gene *ypl*A is part of the flagellar regulon. J Bacteriol.

[B38] Young GM, Schmiel DH, Miller VL (1999). A new pathway for the secretion of virulence factors by bacteria: the flagellar export apparatus functions as a protein-secretion system. Proc Natl Acad Sci USA.

[B39] Young BM, Young GM (2002). YplA is exported by the Ysc, Ysa, and flagellar type III secretion systems of *Yersinia enterocolitica*. J Bacteriol.

[B40] Jacob-Dubuisson F, Fernandez R, Coutte L (2004). Protein secretion through autotransporter and two-partner pathways. Biochim Biophys Acta.

[B41] Warren SM, Young GM (2005). An amino-terminal secretion signal is required for YplA export by the Ysa, Ysc, and flagellar type III secretion systems of *Yersinia enterocolitica *biovar 1B. J Bacteriol.

[B42] Linares JF, Gustafsson I, Baquero F, Martinez JL (2006). Antibiotics as intermicrobial signaling agents instead of weapons. Proc Natl Acad Sci USA.

[B43] Kaufman M, Thomas R (2003). Emergence of complex behaviour from simple circuit structures. C R Biol.

[B44] Rigali S, Schlicht M, Hoskisson P, Nothaft H, Merzbacher M, Joris B, Titgemeyer F (2004). Extending the classification of bacterial transcription factors beyond the helix-turn-helix motif as an alternative approach to discover new cis/trans relationships. Nucleic Acids Res.

[B45] Guespin-Michel JF, Polack B, Merieau A (2003). Bacterial adaptation and epigenesis. Recent research and Developments in Microbiology.

[B46] Filopon D, Merieau A, Bernot G, Comet JP, Leberre R, Guery B, Polack B, Guespin-Michel J (2006). Epigenetic acquisition of inducibility of type III cytotoxicity in *P. aeruginosa*. BMC Bioinformatics.

[B47] Holloway BW, Krishnapillai V, Morgan AF (1979). Chromosomal genetics of *Pseudomonas*. Microbiol Rev.

[B48] Burini JF, Gugi B, Merieau A, Guespin-Michel JF (1994). Lipase and acidic phosphatase from the psychrotrophic bacterium *Pseudomonas fluorescens*: two enzymes whose synthesis is regulated by the growth temperature. FEMS Microbiol Lett.

[B49] Dacheux D, Attree I, Toussaint B (2001). Expression of ExsA in trans confers type III secretion system-dependent cytotoxicity on noncytotoxic *Pseudomonas aeruginosa *cystic fibrosis isolates. Infect Immun.

[B50] Karkhanis YD, Zeltner JY, Jackson JJ, Carlo DJ (1978). A new and improved microassay to determine 2-keto-3-deoxyoctonate in lipopolysaccharide of Gram-negative bacteria. Anal Biochem.

[B51] Youssef NH, Duncan KE, Nagle DP, Savage KN, Knapp RM, McInerney MJ (2004). Comparison of methods to detect biosurfactant production by diverse microorganisms. J Microbiol Methods.

[B52] Laemmli UK, Molbert E, Showe M, Kellenberger E (1970). Form-determining function of the genes required for the assembly of the head of bacteriophage T4. J Mol Biol.

[B53] Deziel E, Comeau Y, Villemur R (2001). Initiation of biofilm formation by *Pseudomonas aeruginosa *57RP correlates with emergence of hyperpiliated and highly adherent phenotypic variants deficient in swimming, swarming, and twitching motilities. J Bacteriol.

[B54] O'Toole GA, Kolter R (1998). Initiation of biofilm formation in *Pseudomonas fluorescens *WCS365 proceeds via multiple, convergent signalling pathways: a genetic analysis. Mol Microbiol.

[B55] West SE, Schweizer HP, Dall C, Sample AK, Runyen-Janecky LJ (1994). Construction of improved *Escherichia-Pseudomonas *shuttle vectors derived from pUC18/19 and sequence of the region required for their replication in *Pseudomonas aeruginosa*. Gene.

[B56] Schnider U, Keel C, Voisard C, Defago G, Haas D (1995). Tn5-directed cloning of pqq genes from *Pseudomonas fluorescens *CHA0: mutational inactivation of the genes results in overproduction of the antibiotic pyoluteorin. Appl Environ Microbiol.

[B57] Simon RPU, Pehle A (1983). A broad host range mobilization system for in vitro genetic engineering: transposon mutagenesis in Gram-negative bacteria. biotechology.

